# Youth in India Ready for Sex Education? Emerging Evidence from National Surveys

**DOI:** 10.1371/journal.pone.0071584

**Published:** 2013-08-09

**Authors:** Niharika Tripathi, T. V. Sekher

**Affiliations:** 1 International Institute for Population Sciences, Deonar, Mumbai, Maharashtra, India; 2 Department of Population Policies and Programmes, International Institute for Population Sciences, Deonar, Mumbai, Maharashtra, India; Midwestern University, United States of America

## Abstract

**Context:**

Sex education/family life education (FLE) has been one of the highly controversial issues in Indian society. Due to increasing incidences of HIV/AIDS, RTIs/STIs and teenage pregnancies, there is a rising need to impart sex education. However, introducing sex education at school level always received mixed response from various segments of Indian society.

**Data and Methods:**

We attempt to understand the expectations and experiences of youth regarding family life education in India by analysing the data from District Level Household and Facility Survey (DLHS-3: 2007–08) and Youth Study in India (2006–07). We used descriptive methods to analyse the extent of access to FLE and socio demographic patterning among Indian youth.

**Results and Discussions:**

We found substantial gap between the proportion of youth who perceived sex education to be important and those who actually received it, revealing considerable unmet need for FLE. Youth who received FLE were relatively more aware about reproductive health issues than their counterparts. Majority among Indian youth, irrespective of their age and sex, favoured introduction of FLE at school level, preferably from standard 8^th^ onwards. The challenge now is to develop a culturally-sensitive FLE curriculum acceptable to all sections of society.

## Introduction

Sex is a very sensitive subject and public discussion on sexual matters is considered as a taboo in Indian society. Given this context, introducing sex education at school level always attracted objections and apprehensions from many quarters. Family life education (FLE) or Sex education refers to a broad programme designed to impart knowledge/training regarding values, attitudes and practices affecting family relationships [1], [2], [3], [4], [5], [6]. It aims to develop the qualities and attitudes on which successful family life depends. The real purpose behind family life/sex education is the transfiguration of a male child into manhood and of a female child into womanhood. The education that provides knowledge on physical, social, moral, behavioural, and psychological changes and developments during puberty is termed as Adolescent Family Life Education. It teaches the adolescents about the role of boys and girls in family and society, responsibility and attitude of boys and girls towards each other, etc. within social context. Many psychologists believe that sex education begins at an early age and continues throughout the life of an individual. The purpose of sex education should be to facilitate the best possible integration between the physical, emotional and mental aspects of the personality, and the best possible assimilation between the individuals and the groups. Sex education also instils the essential information about conception, contraception and sexually transmitted diseases. It is a continuous process of developing attitudes, values and understanding regarding all situations and relationships in which people play roles as males or females [7].

The major objectives of Family Life/Sex Education (FLE) can be broadly described as follows: 1) To develop emotionally stable children and adolescents who feel sufficiently secure and adequate to make decisions regarding their conduct without being carried away by their emotions. 2) To provide sound knowledge not only of the physical aspects of sex behaviour but also its psychological and sociological aspects, so that sexual experience will be viewed as a part of the total personality of the individual. 3)To develop attitudes and standards of conduct which will ensure that young people and adults will determine their sexual and other behaviour by considering its long range effects on their own personal development, the good of other individuals, and welfare of society as a whole [7].

More than biological specifics, sex education should also include social and moral behaviour, proper attitudes and values towards sex, love, family life and interpersonal relations in the society. Due to growing incidences of HIV/AIDS, RTIs/STIs and teenage pregnancies, there is a need to impart sex education among youth. The problem of over-population also demands family life education, including family planning as a priority, as many of the young people are about to be married and should be aware of the responsibilities they have. A study on child abuse in India, conducted by the Ministry of Women and Child Development, reports that 53 percent of boys and 47 percent of girls surveyed faced some form of sexual abuse [8]. Therefore, family life education might help the vulnerable young population to be aware about their sexual rights and empower them to protect themselves from any undesired act of violence, sexual abuse and molestation. India’s National Population Policy also reiterates the need for educating adolescents about the risks of unprotected sex [9]. Furthermore, the provision of family life education might result into multiple benefits to the adolescent boys and girls. This might include delayed initiation of sexual activity, reduction in unplanned and early pregnancies and their associated complications, fewer unwanted children, reduced risks of sexual abuse, greater completion of education and later marriages, reduced recourse to abortion and the consequences of unsafe abortion, curb the spread of sexually transmitted diseases including HIV [10].

Adolescence (10–19 years) is an age of opportunity for children marked with a time of transition from childhood to adulthood; wherein young people experience substantial changes in their physiology after puberty, but do not instantaneously imbibe the various associated roles, privileges and responsibilities of adulthood. This crucial period in the lives of young people presents prospect to promote their development and equip them with appropriate knowledge, attitudes, beliefs and skills (KABS) to help them successfully navigate through various nuisance and vulnerabilities of life, and realize the full development potential [11].

Current statistics indicate that almost one in every fifth person on the globe is an adolescent, as they comprise 18 percent (1.2 billion) of world’s population in 2009, with 88 percent living in developing countries, particularly in the South Asia, the East Asia and the Pacific region [11]. India has the largest adolescent population (243 million), followed by China (207 million), United States of America (44 million), Indonesia and Pakistan (41 million each). Interestingly, more than 50 percent of the adolescent population lives in urban areas, which is expected to further reach 70 percent mark by 2050, with the largest increase likely to occur in the developing world. This entire scenario indicate the considerable demographic and socioeconomic challenges, particularly for the developing countries like India, in terms of meeting the specific needs for improving the survival and general health conditions, nutritional status, and sexual and reproductive health of the adolescents.

Recent literature on adolescents have documented that irrespective of being relatively healthy period of life, adolescents often engage in the range of risky and adventurous behaviours that might influence their quality of health and probability of survival in both short and long term over the life course [12]. These includes early pregnancy, unsafe abortions, sexually transmitted infections (STIs) including HIV, and sexual abuse and violence. Pregnancy related problems comprise a leading cause of death among adolescents aged 15–19 years, mainly due to unsafe abortions and pregnancy complications [13]. However, the sexual and reproductive health needs of adolescents and youth are poorly understood and grossly underappreciated owing to limitation of scientific evidence compounded with the unpreparedness of public health system, which may jeopardize the initiatives to advance the health and well-being of adolescents.

Adolescents and youth in India experience several negative sexual and reproductive health outcomes such as early and closely spaced pregnancy, unsafe abortions, STI, HIV/AIDS, and sexual violence at alarming scale. One in every five woman aged 15–19 years experience childbearing before 17 years of age that are often closely spaced; risk of maternal mortality among adolescent mothers was twice as high as compared to mothers aged 25–39 years [14], [15]. Importantly, adolescents and youth comprise 31 percent of AIDS burden in India [16]. Furthermore, multiple socioeconomic deprivations further increase the magnitude of health problems for adolescents. This limits their opportunity to learn and access the appropriate health care services.

This inadvertent scenario calls for a serious and comprehensive public health initiative to provide Indian adolescents and youth with accurate and age-appropriate essential information and skills for a responsible lifestyle, that might help in reduction of risky sexual behaviour, early pregnancy, HIV/AIDS and STI, etc. Recently, recognizing the need of the time, Government of India has experimented with the provision of Adolescent Education Programme (AEP) to lay the foundation for a responsible lifestyle, including healthy relationships and safe sex habits among adolescents and youth. However, this initiative attracted mixed reactions from different sections of the Indian society. There is scanty scientific literature which throws light on the level of knowledge, perceptions and viewpoints on issues related to family life education among Indian adolescents and youth. Are adolescents and youth in India really prepared to understand and benefit from this new experiment? Hence there is a need for studies that scrutinize and critically evaluate the knowledge, attitude, perceptions, skills and experiences of family life education among Indian adolescents.

### Controversy Over Introducing Sex Education in Schools

With the view to generate awareness and inculcate necessary skills among adolescents and youth, a scheme for adolescent education programme in the school curriculum was promoted by the National AIDS Control Organization (NACO) and the Ministry of Human Resource Development (MHRD), Government of India, which led to a major controversy in 2007. The ardent opponents argued for a ban on starting sex education in schools on the ground that it corrupts the youth and offends ‘Indian values’ [17], [18]. They contended that it may lead to promiscuity, experimentation and irresponsible sexual behaviour [19]. The critics also suggested that sex education may be indispensable in western countries, but not in India which has a rich cultural traditions and ethos. On the contrary, the proponents argued that conservative ideas have little place in a fast modernizing society like India, where attitudes towards sex education are changing rapidly. As fallout of this controversy, several Indian states including Gujarat, Madhya Pradesh, Maharashtra, Karnataka, Kerala, Rajasthan, Chhattisgarh and Goa declared that the course content as suggested by MHRD was unacceptable and thus banned the programme [1].

At the same time, attempt towards the introduction of sex education at school level in India met with opposition from the fundamentalists arguing that it may degrade the tender minds and destroy the rich family systems in India. Furthermore, some teachers and principals were threatened that, “if you don’t stop sex education, neither will you remain in the jobs, nor will your schools survive”. However, the other side of the coin (*pro for sex education*) reflects supportive campaign towards introduction of sex education that may help to reserve the rich heritage and culture of India. Adolescents should be scientifically educated about the facts and myths related to sexual activities that may lead to number of health related risks. Being vulnerable to various changes associated with physical, emotional and psychological transitions, adolescents/youth must have proper knowledge of sex education that may empower them into healthy, productive and responsible adults [20].

Though few politicians and religious leaders have opposed the introduction of sex education in schools, studies have shown that Indian adolescents and youth do not have sufficient information about sexual matters, thereby increasing the possibility of falling prey to various forms of sexual violence. TARSHI (Talking about Reproductive and Sexual Health Issues), a non-governmental organization running a helpline on sexual information, received over 59,000 calls from men, seeking information on sexual anatomy and physiology [1]. An analysis of this data showed that, 70 percent of the callers were below 30 years of age, while 33 percent were in the age group of 15 to 24 years, which indicates that young people do have the need, but lack adequate authentic source to receive appropriate and correct information in a positive manner. The WHO report (2003) on family life, reproductive health and population education documented that promotion of family life/sex education has resulted in delayed age of entering into sexual relationship, reduced number of partners, increased use of safer sex and contraception, and other positive behaviour [10]. It was further noted that sex education in schools did not encourage young people to have sex at earlier age; rather it delays the start of sexual activity and encourages young people to have safer sex. However, both the critiques and proponents of introducing family life/sex education in Indian schools propagate the analogous ideology of ‘sexual restraint’ i.e., delaying the initiation of sexual activity among adolescents before marriage, which may also help to curtail the menace of HIV/AIDS, sexually transmitted diseases and restrict the pace of population growth [21].

India has become the second largest hub of HIV/AIDS pandemic in the world. The proponents of sex education stressed the need for providing knowledge about HIV/AIDS, teenage pregnancies and information about sexual health. In a survey of college students conducted by the All India Educational and Vocational Guidance Association, it was reported that 54 percent of males and 42 percent of females did not have adequate knowledge regarding matters of sex [7]. About 30 percent of males and upto 10 percent of females are sexually active during adolescence before marriage, though social attitudes clearly favour cultural norms of premarital chastity [14].

We need to accept the fact that we are living in a complex world leading complicated lives. Preventing access to pornographic movies or erratic contents on television shows is not prudent, but adding a single chapter to the school curriculum is relatively simple and practical [22]. Mass media being highly influential has been part of both solution and of the problem in the area of sex and youth. It has been part of the solution because it has helped to bring sexual topics into discussions. Radio and television has been the medium in opening doors to the deliberations of several topics which were previously considered as taboo. A survey conducted in Mumbai found that 88 per cent of the boys and 58 per cent of the girls among college students had received no sex education from parents and their source of information were books, magazines, and youth counsellors [7]. Internet is the greatest culprit which makes pornography easily accessible in recent times. Studies have shown that vast majority of parents do not accept the responsibility for providing sex education to their sons or daughters [23]. However, another study states that 68 percent of the parents believe that they should be the primary sex educators of their children, followed by schools [24].The apparent stigma attached to any discussion on sex in India is due to the fact that people tend to view sex education in a narrow sense, that is, the mere explanation of anatomical and biological differences. Ideally home is the best place for sex education and the attitudes of parents are of vital importance. When a child feels the subject as forbidden, he/she feels more curious to know about it which can lead to misleading information, if parents feel embarrassed in talking about sex with their children.

### Available Evidences

The recent emerging scientific evidence across globe documents substantial confirmatory positive influence of sex education towards promoting overall health and well-being of adolescent and youth. A recent study from Nigeria presents paramount significance of providing sexual education to youth that helped them to develop critical thinking and insights on range of family life/sexual issues like premarital sex and pregnancy, abortion, teacher-student relationships and lesbianism [25]. Another study in Indonesia suggests the mixed viewpoint on the pros and cons of sex education among youth [26]. Proper information about sexuality should be provided to youth to help them grow healthy and responsible. A study conducted in Venezuela highlighted the importance of imparting sex education to youth, as it helped to prevent adolescent pregnancy, abortion, HIV/AIDS and sexual abuse [27]. A study in India revealed that majority of school teachers was in favour of imparting sex education to school children [28]. Fourteen years of age was considered to be the most appropriate for imparting sex education by 28.6 percent of school teachers. School teachers and doctors were considered to be the most appropriate persons for providing sex education. Another study from India attempted to assess the impact of sex education on the students noted that doctors were the first choice to impart sex education followed by school teachers; the preferred mean age to start sex education was 15–16 years [29]. A study conducted in seven private co-educational schools to understand the adolescent attitudes towards issues of sex and sexuality in India showed wide lacuna in the knowledge on sex and sexuality matters among adolescents [30].

Majority of mothers believed that discouraging pre-marital intercourse should be the most important objective of sex education, and those who felt that their own sex education was inadequate were in support of providing sex education for their children [31]. Parents should provide sex education to their children in a friendly and informal atmosphere so that children may get rid of the idea that sex is dirty and be aware of their responsibilities [32].

A survey conducted in Hyderabad and Secunderabad cities of India found that the major sources of information on sexual matters among adolescents were books and films, followed by friends [33]. An important observation emerging from this study is that, in spite of exposure towards sex education, many adolescents did not have the correct knowledge regarding reproduction process. This further raises serious questions regarding the content, technique and format of the sex education being imparted in certain institutions which failed to have a desired impact on adolescents/youth. Family life education for boys and girls at the adolescent stage should be constructive enough so as to contribute to healthier emotional growth and it must prepare them to enter into a responsible adulthood [34]. Adolescent boys and girls need sound and correct knowledge about sexual matters. In general, the knowledge among boys regarding sexual issues is more than that of girls may be because boys try to satisfy their curiosity more readily [23]. It was also found that educated parents help their children to clarify their doubts and anxieties about sexual matters in a more realistic way. The findings of National Family Health Survey show that majority of men and women in India favour family life education [35]. More than two-thirds of adults approve of teaching school children about physical changes in their bodies that come with puberty, although there is somewhat less approval of children learning about puberty in the opposite sex.

According to the Youth Study in India [36], 83 percent of young men and 81 percent of young women (aged 15–24 years) felt the need to impart family life education. However, there exists a substantial rural-urban differential in reporting of the need for family life/sex education. Those who received the family life education consisted of only 23 percent of unmarried women and 17 percent of unmarried men.

### Youth Ready for Sex Education?

Though few micro-level studies have been conducted in India to examine the knowledge, attitude and perceptions of adolescents toward family life education, yet there exists huge gap in appropriate understanding regarding various issues of family life/sex education and its effective implementation. Since there are supporters and opponents towards introducing sex education in Indian schools, it is most important to understand the perception and attitudes of youth on this controversial issue. This study is an attempt in the same direction using evidence from two nationally representative sample surveys to analyse the perceptions and experiences of family life education among young women in India. These large-scale household surveys [36], [37] conducted across India and various parts thereof, provide a unique opportunity for the first time to gauge the attitudes of younger generation. In this study, the terms sex education/family life education/adolescent life education were interchangeably used.

The present study broadly attempts to gauge the views, perceptions, aspirations and experiences of adolescents and youth regarding family life education. The specific objectives are as follows:

1. To study the perception regarding family life education (FLE) among adolescents and youth.
2. To examine the experiences of youth who received family life education.
3. To evaluate the awareness on reproductive health (RH) issues among youth and the impact of FLE on their awareness.

### Ethics Statement

The study was based on an anonymous public use data set with no identifiable information on the survey participants; therefore no ethics statement is required for this work.

## Data and Methods

The data for the present analysis comes from two major household surveys in India. The District Level Household and Facility Survey (DLHS-3) [37] in 2007–08 is perhaps the largest ever demographic and health survey carried out in India with a sample size 7,20,320 households covering 601 districts of the country. The perception and knowledge about family life education, family planning, RTI/STI, HIV/AIDS and reproductive health issues were collected in this survey. About 1,60,550 unmarried women were interviewed in DLHS-3, using a structured interview schedule.

The second survey is the “Youth in India: Situation and Needs” conducted in 2006–07 in six Indian states [36]. The main objective of this survey is to gather evidence on key transitions experienced by youth as well as their awareness, attitudes and life choices. The study was conducted in the following selected Indian states: Andhra Pradesh, Bihar, Jharkhand, Maharashtra, Rajasthan and Tamil Nadu. In all, 50,848 married and unmarried young women and men were successfully interviewed, from 1,74,037 sample households. Unmarried men and women as well as married women (15–24 years) were interviewed, whereas the age group for married men was extended to 15–29 years, in the first ever landmark survey on youth in India.

Literature suggests that the attitudes and behaviour of youth are usually influenced by socio-economic, cultural and demographic characteristics. The pertinent socio-economic and demographic characteristics considered in this study includes age groups (15–19 and 20–24 years), type of residence (rural and urban), religion (Hindu, Muslim, Christian and others), caste (Scheduled Caste, Scheduled Tribe, Other Backward Classes and others), education (non-literate, 1–5 years of schooling, 6–9 years and 10 years or above), economic status of the household as presented by wealth index and employment status (not working, agriculture, manual, non-manual). Awareness about contraceptives has been computed based on modern methods (sterilization, pills, condom, IUD, etc.) and traditional methods (rhythm, withdrawal, abstinence, etc.).

### Findings

**Table 1** presents the composite picture concerning the perceived importance of family life education (FLE) and the perception regarding at what age and standard it should be introduced in India. Nearly four-fifths of unmarried women (15–24 years of age) perceived that FLE is important. DLHS-3 asked women about their opinion regarding- at what age and at what level in school does the FLE should be introduced? Majority of women reported that FLE should be provided in the age group 15–17 years (38 percent) and initiated from the 8^th^ to 10^th^ standards (55 percent). The information regarding major sources of FLE among unmarried women who perceived FLE to be important was also collected. Majority of the respondents reported that the main source for providing FLE should be parents (81 percent), followed by teacher/school/college (55 percent), sibling/sister-in-law (50 percent), and friends/peers (30 percent) respectively. On the other hand, health care provider/experts (10 percent), husband/partner (4 percent), youth club/NGO worker (3 percent) were respectively chosen as other preferred sources of information on FLE among unmarried women in India.

**Table 1:** Awareness and perception regarding family life/sex education among Unmarried Women (percentages).

Perceptions	Unmarried Women
**Perceived family life/sex education to be important**	80.9
Family life/sex education should be provided at age (years)*	
Below 12	7.4
12–14	33.2
15–17	37.3
18 or above	17.7
Family life/sex education should be provided from standard*	
Below 8	22.7
8–10	54.9
Above 10	15.0
The best person to impart family life/sex education*	
Parent	81.3
Sibling/sister-in-law	49.9
Spouse/partner	3.9
Teachers in School/College	55.1
Friends/Peer	29.5
Health care provider/expert	9.6
Youth club/NGO worker	3.5
**Total number of women interviewed in the survey**	1,60,550

Note: *Among Women who perceived family life education to be important.

Source: - IIPS, 2010.

**Table 2** indicates the proportion of women who actually received FLE and their experiences regarding the same. Around 50 percent of women actually received FLE, overwhelming majority from schools or colleges. The other sources were NGO programmes, youth clubs, government programmes, etc. Among the women who received FLE, majority reported that the teacher/trainee explained it in a way that can be understood and FLE answered/clarified many of their questions. It is important to note that, around 40 percent of women felt embarrassed while attending family life/sex education classes.

**Table 2:** Experiences of Unmarried Women who received family life/sex education (percentages).

Experiences	Unmarried Women
**Received family life/sex education**	48.5
**Source of family life/sex education**	
School/College	78.9
Youth club	6.7
Government programme/camp	5.4
NGO programme/camp	3.7
Others	24.4
**Opinion about family life/sex education received**	
It answered many queries	79.6
Teacher/trainer explained well	83.7
Respondent felt embarrassed	40.8
**Number of women who received family life/sex education**	74,475

Source: IIPS, 2010.

**Table 3** presents the percentage of unmarried women 15–24 years who perceived FLE to be important, and those who actually received FLE by selected demographic and socioeconomic characteristics in India. The prevalence of perceived importance of FLE was relatively high among the youth (81 percent) in India. However, only 49 percent of women actually received FLE. The relatively mature unmarried women (20–24 years) residing in urban areas with more than ten years of education, engaged in non-manual occupation, and coming from better-off families had higher prevalence of perceived importance of FLE as well as that of receiving FLE than others. In general, the perceived importance of FLE among youth in India is relatively high with strong demographic and socioeconomic differentials. The actual experience of FLE among youth is extremely limited.

**Table 3:** Perception and actual experience of family life/sex education among unmarried women by their background characteristics (percentages).

Background Characteristics	Perceived family lifeeducation is Important	Received familylife education	Number of Women
**Age Group**			
15–19	78.7	45.9	120,586
20–24	86.8	55.7	39,965
**Residence**			
Rural	77.4	43.4	117,428
Urban	85.1	54.8	43,123
**Present level of Education of Women** **Women**			
Non Literate	50.8	16.0	15,269
1–5 years	61.8	21.1	19,226
6–9 years	79.5	42.9	59,900
10 years or above	92.0	65.5	66,156
**Employment Status**			
Not working	82.5	51.3	113,977
Agriculture	71.3	34.6	25,462
Manual	75.3	38.0	13,612
Non-manual	88.3	60.0	7,500
**Religion**			
Hindu	81.1	48.5	113,754
Muslim	77.9	42.6	20,812
Christian	81.3	57.4	12,817
Others	83.3	51.9	13,168
**Caste/Tribe**			
Scheduled Caste	79.3	44.9	26,577
Scheduled Tribe	73.9	44.8	32,669
Other Backward Classes	79.7	46.7	56,703
Others	87.3	55.0	44,602
**Wealth Index**			
Lowest	61.2	25.1	17,955
Second	70.2	32.8	23,948
Middle	75.8	40.6	32,823
Fourth	82.6	50.2	40,748
Highest	90.1	61.9	45,077
**India**	**80.9**	**48.5**	**160,550**

Source: IIPS, 2010.

The knowledge and awareness on reproductive health issues among unmarried women were also collected in the DLHS-3. On an average, the women who received FLE had much better awareness on various reproductive health issues like RTI/STI, possibility of finding out the sex of a baby before birth, and knowledge about reducing chances of infecting HIV as compared with women who did not receive any FLE (**Table 4**). In general, women who received the FLE were relatively more aware about methods of contraception as compared to their counterparts. For instance, among women who received FLE, nearly 98 and 27 percent of women were aware about any modern and traditional methods of contraception respectively. On the other hand, this figure declines to 89 and 12 percent respectively among women who do not receive FLE.

**Table 4:** Knowledge and Awareness on Reproductive Health Issues among Unmarried Women, with and without FLE (percentages).

Reproductive Health Issues	Women received familylife/sex education	Women did not receive familylife/sex education
**Awareness regarding RTI/STI**	46.0	20.9
**Possible to know the sex of the baby before the baby is born**	67.8	57.4
**Knowledge about how to reduce the chances of infecting HIV** ^φ^	79.9	49.8
**Knowledge on contraceptives**		
Any modern method#	97.6	89.8
Any traditional methodΨ	26.7	12.2

Note: ^φ^Knowledge about how to reduce HIV/AIDS transmission includes the awareness of the respondents on any of the ways to reduce the chances of getting HIV/AIDS such as abstain from sex, using condoms correctly, limit sex with one partner/stay faithful to one partner, limit number of sexual partners, avoid sex with sex workers, avoid sex with persons who have many partners, avoid sex with homosexuals, avoid sex with persons who inject drugs, use tested blood, use only new/sterilized needles, avoid IV drip, avoid sharing razors/blades, avoid pregnancy when having HIV/AIDS.

# Any modern method of contraception includes female sterilization, pill, condom/nirodh, male sterilization, IUD, injectables and emergency contraceptive pills.

Ψ Any traditional method of contraception includes rhythm, withdrawal and other traditional methods.

Source: IIPS, 2010.

**Table 5** illustrates the young people's opinion on family life/sex education across men and women, and married and unmarried. Around 83 percent of young men and 78 percent of young women felt that it is important to impart family life/sex education to youth. Slightly large proportion of unmarried youth (84 percent of men and 81 percent of women) as compared to married youth (79 percent of men and 75 percent of women) reported family life/sex education to be important. Majority of young men and women observed that family life/sex education should be provided to adolescents in the age group 15–17 years. Regarding the perception of youth about the best person to impart family life/sex education, the preferences differed among men and women. Majority of young men reported that the best person to provide FLE should be teacher, whereas most young women suggested that parents are ideal persons to provide such education. Around 21 percent of young men and 11 percent of young women reported that the main source of providing family life/sex education can be friends.

**Table 5:** Perceptions of Youth regarding Family Life Education (percentages).

Perceptions	Men	Women	MarriedMen	MarriedWomen	Unmarried Men	Unmarried Women
**Perceived family life/sex** **education to be important**	82.5	78.1	79.5	75.5	83.7	81.0
**Family life/sex education should be provided at age (years)**						
Below 12	2.8	2.6	3.3	3.2	2.7	2.1
12–14	12.1	23.6	10.7	22.6	12.6	24.7
15–17	47.5	42.6	41.7	41.5	48.7	43.7
18 or above	35.4	25.5	41.4	25.6	34.3	25.2
**The best person to impart family life/sex education is**						
Parent	5.9	33.8	7.4	36.7	5.7	30.4
Sibling/sister-in-law	0.4	5.6	0.4	6.5	0.4	4.8
Spouse/partner	0.2	2.4	0.3	4.2	0.2	0.3
Teacher	44.8	27.3	36.7	20.2	47.7	34.9
Friend	21.2	11.4	23.7	11.6	20.2	11.3
Health care provider/expert	23.2	14.5	27.1	14.7	21.9	14.2
Youth club/NGO worker	0.9	0.4	0.9	0.3	1.0	0.4
Total sample	14,281	31,274	8,052	13,912	11,522	17,362

Source: IIPS & Population Council, 2010.

**Table 6** indicates that, nearly 15 percent of young men and 14 percent of young women received family life/sex education. Majority received FLE through schools/colleges. Among those who received formal family life/sex education, majority felt that FLE answered many of their anxieties/queries and the teacher/trainee explained the subject well. Twenty one percent of men and 37 percent of women also reported that they felt embarrassed while attending family life/sex education. This, in a way suggests that the curriculum and the method of teaching should be context-specific and culturally sensitive.

**Table 6:** Experiences of Youth who received Family Life Education (percentages).

Source/Experiences	Men	Women	Married Men	MarriedWomen	Unmarried Men	Unmarried Women
**Those who received family life/sex education**	15.3	14.6	7.8	7.3	17.4	23.0
**Source of family life/sex education received**						
School/College	80.0	87.9	64.5	77.1	81.6	91.8
Government programme/camp	16.3	11.8	26.2	21.2	15.3	8.3
NGO programme/camp	6.4	5.1	13.6	8.9	6.1	3.7
**Opinion about family life/sex education received**						
It answered many queries	87.4	90.0	89.7	91.7	87.1	89.3
Teacher/trainer explained well	89.8	91.2	84.8	91.0	89.6	91.3
Respondent felt embarrassed	21.3	36.6	19.3	36.7	21.1	36.6
Total number of youth who received family life/sex education	2,061	4,219	651	968	1,884	3,251

Source: IIPS & Population Council, 2010.

## Discussion and Conclusion

The present study attempt to unravel the divergent views, perceptions and aspirations of adolescents and youth regarding family life education, its perceived importance, and the potential effects of family life education on array of reproductive health issues by using nationally representative household sample surveys in India. Young people (10–24 years) constitute about 315 million and represented about 31% of India’s population. They not only represent India’s future in the socio-economic and political realms, but nation’s ability to harness the demographic dividend. In the course of transition to adulthood, young people face significant risks related to sexual and reproductive health. Adolescent life education program intend to ensure the rights of the large section of adolescents/youth, and to develop them as healthy and responsible members of the family and society. Adolescents in all societies learn their responsibilities towards family by observing and following the behaviour of others. Due to rapid social changes occurring all over the world, the young generation is facing an enormous challenge in coping with the consequences of attrition in the traditional family system, social life, and values. Under this volatile environment, the family life education can help the adolescents to experience successful transition from childhood to adulthood.

One of the most significant findings of the study indicates that majority of youth perceived family life education to be important. This highlights that Indian adolescents realizes the range of potential health risks and challenges lurking before them and demands the appropriate knowledge, skills and training to lead a responsible and healthy life style. However, the study points out that only half of the unmarried women actually received any form of family life education. This critical mismatch between the potential demand for FLE and apparent lack of facility might lead to a brigade of untrained, ill-equipped and unmanageable group of risky young population who might indulge into unhealthy life style practices. Another finding that needs special attention relates to the appropriate age and standard at which the FLE should be initiated. The findings based on the opinions of unmarried women 15–24 years suggest that the FLE should be started at the age of 15 to 17 years and during 8^th^ to 10^th^ standard in schools.

One of the crucial issues that deem attention relates to the major sources of FLE. The study indicates that majority of unmarried women, who perceived FLE to be important, reported that parents to be the provider of FLE, followed by teacher/school/college, brother/sister/sister-in-law and friends/peer respectively. Therefore, it becomes apparent that FLE need not be only part of formal school curriculum; it should also be equally augmented in the first place by parents at home to eliminate all the misconceptions, inhibitions and doubts of adolescents on various aspects of family/sex life. The study also indicates that relatives and friends/peers could also be important avenues that need to be appropriately tapped to help the adolescents learn about the basic issues/rules of family/sex life skills safely and comfortably either at home, school or neighbourhood.

Addressing the discourse on the implementation of FLE in school curriculum in India, several scholars, administrators, and politicians have mooted the adverse impact of FLE and how it may denigrate the ‘rich Indian cultural values’ and ethos. However, our findings effectively nullifies all these apprehensions and convincingly illustrates that, among youth who received FLE, the awareness about various reproductive health issues and knowledge of contraceptive methods was far better and comprehensive compared to their counterparts who had no FLE. This further goes on to show that provision of FLE will benefit not only the adolescents, but many more generations to come by avoiding the menace of RTI/STI, unwanted pregnancies, HIV/AIDS, etc.

In the era of globalization and modernization, there still persist steep socioeconomic divide in the knowledge, attitudes and perception of individuals in Indian society. The same holds true with regard to benefits of FLE. Whether it relates to the perceived importance of FLE, or actual prevalence of FLE among unmarried women in India, we found substantial differentials across socio-economic groups. This indicates that even after more than six decades of planned development efforts in India, large proportion of population living in rural areas, illiterate, marginalized social groups, continue to lag behind when it comes to the adoption of modern attitudes and healthy sexual behaviour. Hence, it is crucial for policy makers and program managers to take note of these socioeconomic hierarchies in Indian society, while designing and implementing any FLE program.

However, most political and religious leaders in India are unfortunately not in favour of sex education at school level. The Rajya Sabha of Indian Parliament constituted a committee to examine the implementation of the Adolescent Education Programme (AEP). The committee categorically opined against the implementation of sex education at school level, and felt that AEP may cause irreparable damage to the future of India by polluting the young and tender minds, and could invariably promote promiscuity. The report also took serious objections on the study materials and kits prepared for the implementation of the AEP in Indian schools. The report while denouncing the case for the implementation of any AEP at school level emphasized the need for appropriately passing on the message of no sex before marriage among adolescents and declaring it as immoral, unethical and unhealthy. In addition, students should be made aware of marriageable age which is 21 years in case of boys and 18 years in case of girls and any indulgence in sex outside the institution of marriage was against the social ethos [38].

Finally, we summarize the key issues that emerge from this study. There exists a wide gap between the proportion of women who perceive FLE is important and those who actually received any sex education. It was also true that women who received family life education had better knowledge and awareness on reproductive health issues than counterparts. The level of awareness and knowledge regarding Family Life Education is more among the educated, better-off sections and those living in urban areas. The growing population, changing life styles and increasing incidences of HIV/AIDS is a great challenge. In order to prepare the youth to face these challenges, introducing sex education is an important step. The nation-wide surveys clearly illustrate that overwhelming majority of young women and men are in favour of introducing family life education. The government and civil society should initiate a national debate to arrive at a consensus on this issue among various sections of the society. The study strongly argues the necessity to formulate appropriate policy regarding family life education so as to address the unmet need for scientific learning/training on matters of family/sexual life among adolescent/youth. Imparting sex education in an appropriate and culturally-sensitive manner at school level should be a national priority.
